# A Personalized Gait Parameter Prediction-Based Speed-Adaptive Control Method for Hybrid Active-Passive Intelligent Prosthetic Knee

**DOI:** 10.3390/biomimetics11020136

**Published:** 2026-02-12

**Authors:** Xiaoming Wang, Yuanhua Li, Hui Li, Shengli Luo, Hongliu Yu

**Affiliations:** 1 School of Health Science and Engineering, University of Shanghai for Science and Technology, Shanghai 200093, China; wangxm@usst.edu.cn (X.W.);; 2 Shanghai Engineering Research Center of Assistive Devices, Shanghai 200093, China

**Keywords:** prosthetic knee, gait parameter prediction, personalized speed adaptation, hybrid actuation, fuzzy logic control

## Abstract

To address the limitations of current prosthetic knees that lack personalized adaptability to users’ gait characteristics and walking speeds, this study proposes a personalized gait parameter prediction–based speed-adaptive control method for a hybrid active–passive intelligent prosthetic knee (HAPK). The proposed system integrates a perceptron-based model to predict individualized gait parameters by mapping anthropometric data and walking speed to key points of the knee trajectory. A fuzzy logic–based damping control for the swing phase and a position–torque control for the stance extension phase are developed to achieve real-time adaptation to different walking speeds and user-specific biomechanics. The hybrid actuation system combines hydraulic damping and motor torque assistance to ensure both compliance and power delivery across gait phases. Experimental results from variable-speed walking tests demonstrate that the proposed control method improves gait symmetry indices—reducing stance and swing asymmetries by approximately 30–38%—and achieves smoother, more natural gait transitions compared to traditional fixed-gait control strategies. These findings validate the effectiveness of the proposed approach in achieving continuous, personalized, and speed-consistent gait control for intelligent prosthetic knees.

## 1. Introduction

Lower-limb amputation often results in significant gait asymmetry, reduced mobility, and decreased quality of life for amputees [1]. The prosthetic knee, as the core component of lower-limb prostheses, plays a crucial role in restoring natural gait and ensuring walking stability. However, due to variations in residual limb conditions, anthropometric parameters, and walking habits, achieving gait coordination that matches the physiological knee motion across amputees remains a major challenge [2]. Therefore, developing prosthetic knees with adaptive and personalized control capabilities has become an important research focus in the field of intelligent prosthetics [3].

Traditional passive prosthetic knees rely on mechanical damping, which can provide basic support and safety during stance but lacks adaptability to speed variations and user-specific gait features [1,2,3,4]. With the advancement of sensing and embedded control technologies, semi-active and active prosthetic knees have been developed to enable variable damping and torque assistance [5,6,7,8,9]. Among these, hybrid active–passive prosthetic knees (HAPK) combine the advantages of hydraulic damping and motor actuation, allowing both energy dissipation and active assistance, and are increasingly used to improve comfort and stability during walking [10,11,12,13,14,15]. Nevertheless, achieving smooth and individualized coordination between damping modulation and torque generation under variable walking speeds poses a complex control challenge.

To enhance gait coordination and comfort, researchers have proposed various speed-adaptive control methods for prosthetic knees. Some studies employ discrete speed switching based on threshold-based control, where damping parameters are adjusted within predefined speed levels [16,17,18]. This approach can provide basic functional adaptation across different walking speeds, yet it generally applies the same parameter settings to all users, without considering individual anthropometric differences. As a result, these systems lack true personalization and often exhibit limited adaptability when facing user-specific gait patterns.

Other approaches employ phase-based impedance, damping, or torque adjustment, in which gait phases are recognized through inertial or plantar pressure sensors, and the corresponding control parameters are modulated in real time [19,20,21,22]. Although this improves dynamic responsiveness, the control parameters are typically determined empirically, without accounting for the complex coupling between anthropometric parameters and gait variations, making it difficult to realize individualized adjustment for different users. Additionally, fuzzy control and neural-network-based models have been explored to establish the relationship between walking speed and damping or assistive torque, enhancing control intelligence [23,24,25,26]. However, most of these models are still based on empirical gait segmentation or single control rules and fail to adequately account for the influence of anthropometric differences on gait characteristics. Consequently, limitations remain in achieving continuous and truly personalized gait prediction.

In response to these limitations, this study proposes a personalized gait parameter prediction-based speed-adaptive control method for a hybrid active–passive prosthetic knee (HAPK). The method establishes a mapping between anthropometric parameters, walking speed, and gait trajectory key points through a perceptron-based prediction model, enabling the generation of individualized gait parameters. On this basis, a fuzzy logic swing-phase damping adjustment and a position–torque stance-extension control strategy are developed to realize continuous adaptation to different walking speeds and user characteristics. Compared with conventional fixed-gait and phase-based control strategies that rely on predefined parameters, as well as learning-based approaches that emphasize speed adaptation without explicit anthropometric personalization, the proposed framework bridges these gaps by integrating gait parameter prediction with hybrid actuation in a unified control architecture. By explicitly incorporating anthropometric characteristics and walking speed into the generation of reference gait parameters, the proposed method enables continuous, personalized, and speed-consistent control across gait phases. In addition, the proposed hybrid actuation and control strategy is bio-inspired by human knee biomechanics during walking, reproducing phase-dependent damping/assistance and speed-consistent knee kinematics to generate more human-like knee trajectories across different walking speeds. By combining predictive modeling with hybrid actuation, the proposed approach aims to achieve more natural, symmetric, and speed-consistent gait performance, thereby improving prosthesis–human coordination and walking comfort.

## 2. Prosthesis Prototype

This section describes the mechatronic design, embedded system and hybrid actuating rules of the HAPK used for the validation of the proposed personalized gait parameter prediction-based speed-adaptive control method.

### 2.1. Mechatronic Design

**Figure 1:**
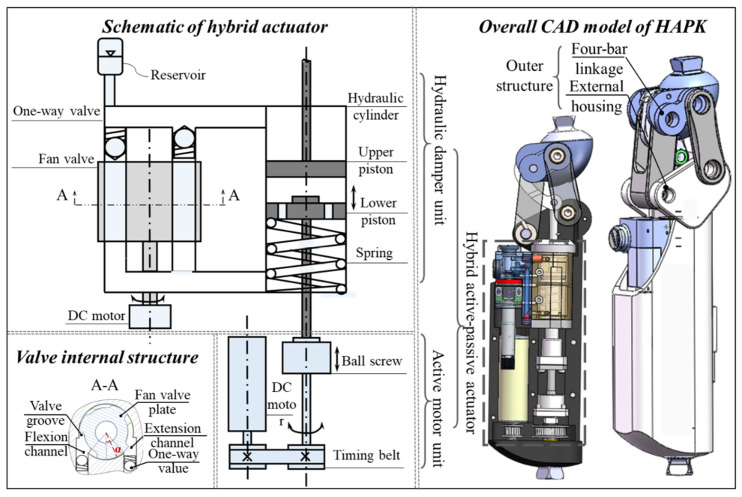
Mechanical schematic of the HAPK.

**Figure 2:**
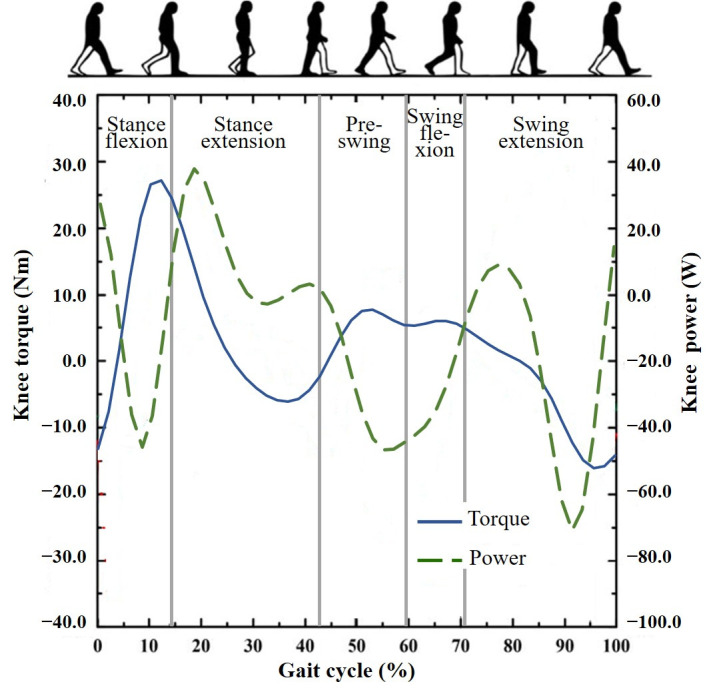
Knee torque and power during a typical gait cycle when walking on level ground.

The proposed HAPK is developed based on our previous work [10,24]. It integrates a hybrid hydraulic–motor actuator that combines the advantages of hydraulic compliance damping and motor-driven torque generation to reproduce the biomechanical behavior of a healthy knee. The overall configuration of the HAPK is shown in Figure 1, consisting of an outer structural frame and a hybrid actuator. The actuator is composed of two primary units: (1) Hydraulic Damping Unit (HDU)—includes a hydraulic cylinder and an electrically controlled fan valve, both developed in-house, capable of dynamically adjusting impedance during the gait cycle; (2) Active Motor Unit (AMU)—consists of a DC motor (Maxon Motor AG, Sachseln, Switzerland) and a transmission mechanism that supplies positive energy to the knee joint when necessary. The HDU adopts a dual-piston coordinated mechanism, where the lower piston is mechanically linked to the AMU. The main design characteristics are summarized in Table 1. The maximum knee torque of 30 Nm was selected with reference to normative level-walking data, where the peak net knee extension moment is typically on the order of 0.4–0.6 Nm/kg. Thus, 30 Nm corresponds to the peak torque demand of an approximately 50–75 kg adult during level walking. Importantly, the proposed HAPK is not intended to fully reproduce the entire biological knee torque profile in the stance phase; rather, the AMU provides assistive positive torque to reduce the user’s effort, while part of the propulsion can still be contributed by the residual limb and proximal joints (e.g., hip). In this hybrid architecture, the AMU mainly assists in the stance extension phase, whereas the HDU supplies adaptive damping in phases dominated by stability and negative work, as further illustrated by the normative knee torque–power profile presented later in Figure 2.

**Figure 3:**
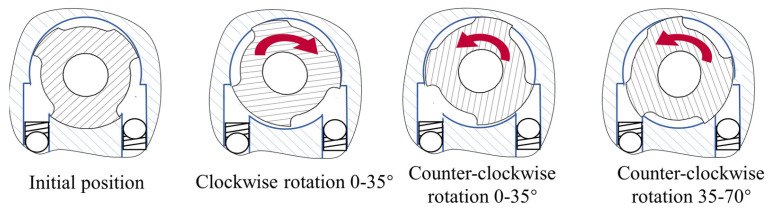
Schematic of typical states of the rotary valve.

In the active mode, the hydraulic damper is set to minimum resistance, and the AMU drives the lower piston upward to deliver positive torque for knee extension. In the passive mode, the AMU is decoupled, and the HDU regulates the hydraulic flow to provide adaptive damping. The rotary valve continuously adjusts the overlap area between the oil channels and valve grooves, allowing a single motor to independently and continuously regulate both flexion and extension damping. As illustrated in Figure 3, the valve operates in four characteristic states during rotation. In the initial position, both flexion and extension damping are minimal. A clockwise rotation of approximately 0–35° increases extension damping while flexion damping remains low. When the valve rotates counter-clockwise by 0–35°, flexion damping increases with little effect on extension. Further counter-clockwise rotation between 35° and 70° gradually closes both oil channels, resulting in maximum damping for both flexion and extension. More details about the novel valve structure design and the hydraulic damping numerical model can be found in [24].

**Figure 4:**
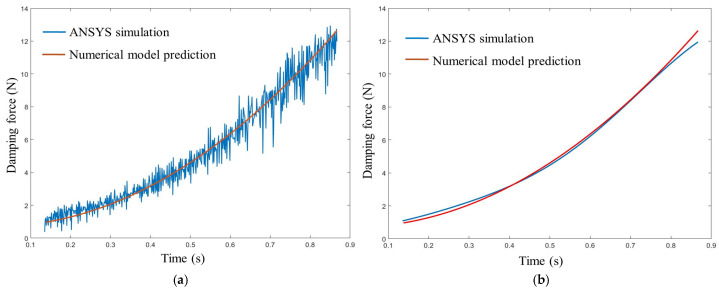
Validation of the hydraulic damping model in swing flexion. (**a**) Comparison between the numerical model prediction and the ANSYS CFD result; (**b**) Comparison with the fitted ANSYS force curve (overall error ≤ 5%).

To further support the credibility of the hydraulic damping model adopted in this study, we performed a swing-phase verification by comparing the damping force predicted by the numerical hydraulic model with the result obtained from CFD-based hydraulic simulation in ANSYS 12.0 under identical conditions. As shown in Figure 4, the analytical prediction closely matches the ANSYS simulated damping-force profile in the swing flexion phase. Moreover, after fitting the force curve and comparing it with the theoretical prediction, the overall error remains within 5%, confirming that the proposed damping-force formulation can reliably approximate the fluid-dynamics-based result for the swing-phase damping behavior.

### 2.2. Embedded System

**Figure 5:**
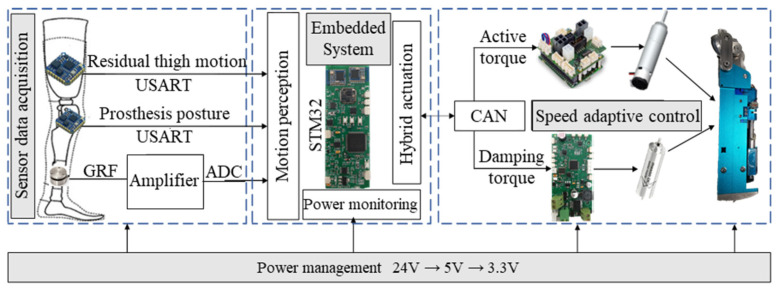
Embedded system of the prosthesis.

The control platform is built around an STM32F407 microcontroller and consists of three main modules: a sensor data acquisition module, an actuator control module, and a power management module, as shown in Figure 5.

The data acquisition module collects information from a load cell and two inertial measurement units (IMUs). These sensors respectively measure the ground reaction force (GRF), the motion of the residual thigh, and the posture of the prosthesis. The acquired signals are transmitted to the main controller via ADC and USART interfaces for processing. The actuator control module drives two brushless DC motors that regulate the hydraulic damping torque and provide active driving torque. Communication and closed-loop control between the controller and the motors are implemented via CAN, ensuring real-time coordination and stable performance. To guarantee the reliable operation of components with different voltage levels, a multi-stage power supply strategy is adopted. The sensors are powered by a 7.4 V lithium battery regulated down to 5 V, the microcontroller operates at 3.3 V, and the motors are powered independently by a 24 V battery to minimize electrical interference and ensure stable performance across the system. To evaluate real-time feasibility on the STM32F407 controller, we measured the computational load of the control loop. Using an on-board cycle counter, the average loop execution time was 0.32 ms. At a loop rate of 1 kHz, this corresponds to an estimated average CPU utilization of 32%, indicating that the embedded implementation can sustain real-time operation with sufficient computational margin.

### 2.3. Hybrid Actuating Rules

Normal human walking indicates that the knee behaves as a hybrid actuation mechanism involving both energy dissipation and energy injection. As shown in Figure 2, knee power is negative in most phases of a typical level-walking gait cycle, whereas positive power is mainly required during stance extension [25,26]. Therefore, providing active torque assistance in stance extension is necessary to help the prosthetic knee extend after stance flexion. Without this power source, reproducing healthy knee behaviors becomes limited, and transfemoral amputees may compensate with more asymmetric gait patterns.

**Figure 6:**
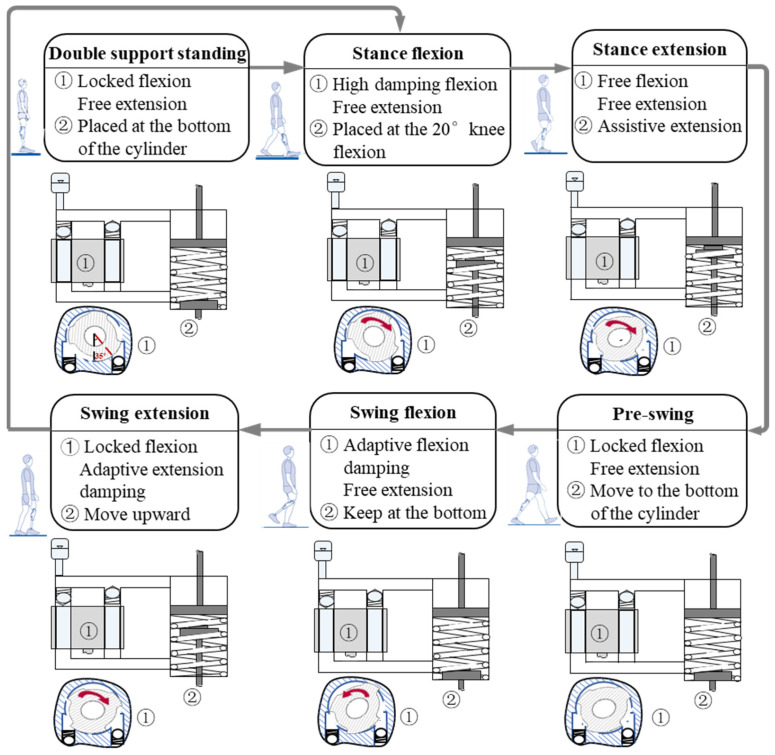
Hybrid actuation scheme for each gait phase during level walking. ① denotes the fan valve in the hydraulic damping unit; ② represents the lower piston connected to the active driving unit.

According to level-walking gait characteristics, one gait cycle is divided into five phases: stance flexion, stance extension, pre-swing, swing flexion, and swing extension. The corresponding hybrid active–passive actuating rules for each phase are summarized in Figure 6. During stance flexion, stability is prioritized; thus, flexion damping is set high while extension damping remains minimal, and the lower piston position limits knee flexion within 20° for safe weight-bearing. During stance extension, the damper is set to minimum resistance and the AMU provides extension torque to follow the desired knee trajectory, enabling smooth stance recovery. During pre-swing, the knee is unloaded and mainly driven by the residual limb; therefore, both damping channels are fully opened to minimize resistance, and the lower piston moves rapidly to the bottom of the cylinder to decouple the active and damping modules. During swing flexion and swing extension, hydraulic damping is adaptively regulated to support smooth and natural knee motion, thereby achieving personalized gait adaptation.

## 3. Control Method

Achieving accurate trajectory tracking of a prosthetic knee according to each amputee’s physical characteristics and walking speed requirements remains a major challenge in prosthetic control. In this study, gait parameter features of subjects with different anthropometric parameters under varying walking speeds were analyzed to establish a perceptron-based gait parameter prediction model. This model predicts the target gait parameters of an amputee based on their individual body parameters and current walking speed. Furthermore, a fuzzy logic–based damping adjustment method for the swing phase and a position–torque control method for the stance extension phase are proposed to achieve gait adaptation across different walking speeds and individual characteristics.

**Figure 7:**
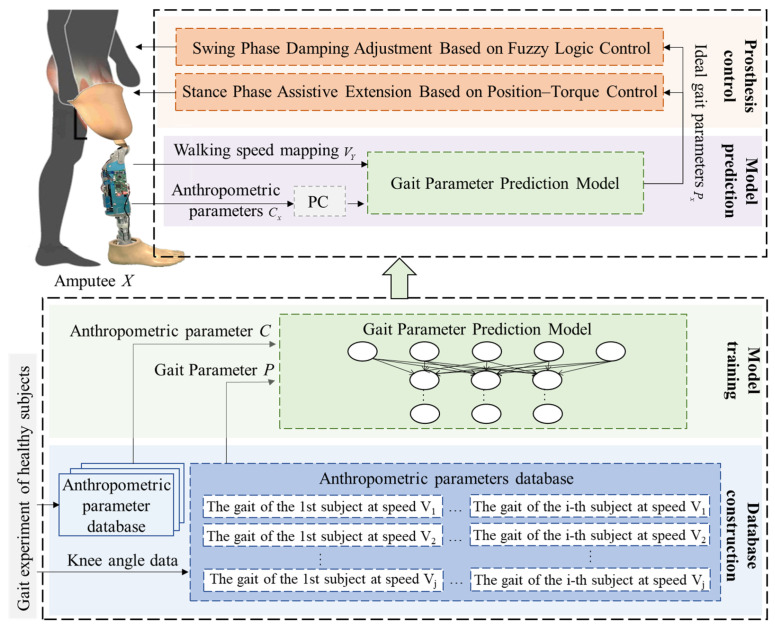
Personalized speed-adaptive control method based on gait parameter prediction.

As shown in Figure 7, the proposed personalized speed adaptive control method consists of four main components: database construction, model training, model prediction, and prosthesis control. Gait and anthropometric data are first collected through variable-speed walking experiments to build the databases. The gait parameter prediction model is trained offline using these data. During real-time walking, the amputee’s parameters and the detected walking speed are used as model inputs to predict the desired gait parameters. These predicted parameters are then used as control targets, with corresponding control strategies applied in different gait phases to fit the predicted gait trajectory adaptively.

### 3.1. Gait Database Construction

Assume that the subject participating in the gait data acquisition experiment is represented as X, and the anthropometric parameters are represented as $C_{x}$. The gait parameter prediction model will predict the gait parameters $P_{x}$ to be fitted for the amputee based on the measured anthropometric parameter information $C_{x}$ and the preset walking speed $V_{y}$. The gait data in the gait database are collected from healthy subjects who have no diseases that affect their walking ability. Assume that the anthropometric parameter database $C\in R^{M\times S}$ is represented as

(1) $$C={\left\{c_{s,1},c_{s,2},\ldots ,c_{s,M}\right\}}_{s=1}^{S}$$

where *M* is the number of body parameters related to gait parameters, and *S* is the number of subjects.

Assume that $D\in R^{L\times S\times I}$ represents the knee motion trajectory of the subject collected at different walking speeds, then

(2) $$D={\left\{\theta_{1,i},\theta_{2,i},\ldots ,\theta_{s,i}\right\}}_{y=1}^{Y}$$

where $\theta_{s,i}\in R^{L}$ is the knee joint angle, *L* is the length of a single gait data collection, and *Y* is the number of walking speeds.

Assume that $\theta_{s,i}\in R^{L}$ represents the gait parameter, then

(3) $$P=\left\{p_{1},p_{2},\ldots ,p_{J}\right\}$$

where *J* is the dimension of gait parameters, that is, the number of key points of the gait trajectory.

A total of 25 healthy participants with heights ranging from 160 cm to 180 cm and no gait impairments were recruited for the experiments. Accordingly, S = 25 in Equation (1) denotes the number of subjects included in the dataset. Seven anthropometric parameters were measured, including height, thigh length, shank length, ankle height, foot length, and the maximum circumferences of the thigh and shank. These data were used to construct the anthropometric database, and their mean and standard deviation values are listed in Table 2.

Gait data were captured using a three-dimensional motion analysis system (MyoMotion, Noraxon, Scottsdale, AZ, USA). IMU sensors were attached to the subjects’ thighs and shanks to record knee joint angles while walking on a treadmill at speeds ranging from 2.0 km/h to 6.0 km/h in 0.5 km/h increments. Each subject walked continuously for 4 min at each speed to ensure sufficient steady-state gait cycles.

During data processing, the initial and final five steps of each trial were removed to eliminate transient adaptation effects. The gait cycles were then segmented according to kinematic features, and key gait parameters (temporal and angular characteristics of critical trajectory points) were extracted to form the gait database.

### 3.2. Gait Parameter Prediction Model

The perceptron model was adopted as a lightweight regression approach to capture the dominant nonlinear relationship between anthropometric parameters, walking speed, and a limited set of gait trajectory key points, rather than for high-dimensional pattern recognition. Moreover, each subject contributed multiple gait cycles across multiple walking speeds, substantially increasing the effective number of training samples at the gait-parameter level. This design supports stable training while maintaining real-time feasibility on embedded hardware. The gait parameter prediction model is constructed using a three-layer perceptron to establish the mapping relationship between the current walking speed and the key point parameters of the gait trajectory for different anthropometric parameters. Previous studies have shown that the length and dimensional parameters of the lower limbs have a significant influence on gait characteristics [27]. Therefore, in this study, seven anthropometric parameters related to body length and dimension—height, thigh length, shank length, ankle height, foot length, and the maximum circumferences of the thigh and shank—were selected. Furthermore, the correlations between these anthropometric parameters and gait trajectory parameters under different walking speeds were analyzed.

**Figure 8:**
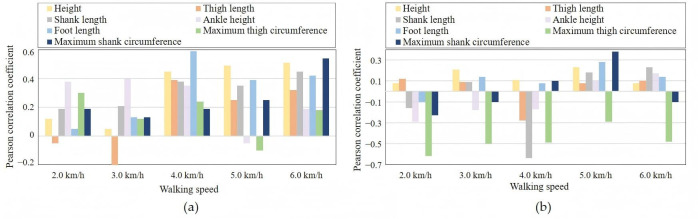
Pearson correlation between anthropometric and gait parameters. (**a**) anthropometric parameters and gait cycle duration; (**b**) anthropometric parameters, maximum knee flexion angle in swing phase.

The Pearson correlation coefficient was used to analyze the relationships between each anthropometric parameter and two important gait trajectory parameters, namely the gait cycle duration and the maximum flexion angle during the swing phase, as shown in Figure 8. From Figure 8, it can be seen that the relationship between anthropometric parameters and gait parameters changes continuously under different walking speeds. For example, the maximum flexion angle during the swing phase shows a negative correlation with most anthropometric parameters at lower walking speeds, while it becomes positively correlated with most anthropometric parameters at higher walking speeds. The gait cycle duration shows a negative correlation with most anthropometric parameters across all walking speeds, which agrees with the general observation that taller individuals tend to have a longer stride length and lower cadence.

Therefore, it can be concluded that the relationship between gait parameters and anthropometric parameters is nonlinear and complex under dynamically changing walking speeds. Thus, employing a neural network model to learn and fit this relationship can better reproduce the normal motion trajectory of a human knee joint [28]. Although the selected length- and dimension-related anthropometric parameters provide a practical basis for personalization, gait is also affected by subject-specific musculoskeletal and soft-tissue factors (e.g., joint laxity, muscle mass/strength distribution, and limb compliance) that are not captured by static measurements. Therefore, the proposed approach should be regarded as an initial personalization step based on easily measurable morphology. Future work will integrate dynamic and neuromuscular information (e.g., EMG) and additional wearable sensing (e.g., IMU-derived limb dynamics or socket-interface signals) and will explore online adaptation to further improve individualization and robustness.

**Figure 9:**
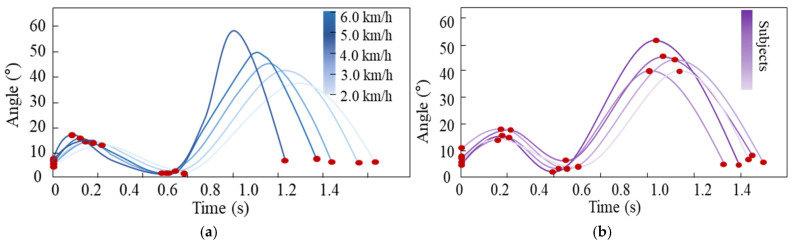
Variations in gait trajectories. (**a**) knee trajectory changes in the same subject at different walking speeds; (**b**) knee trajectory differences among different subjects at the same walking speed (4 km/h).

The gait parameter prediction model can predict gait parameters $P_{x}$ according to the anthropometric parameters and the actual walking speed. However, selecting appropriate gait parameters—so that the gait trajectory can be represented by only a few key points while still achieving smooth and natural gait motion—is one of the key issues. Figure 9a,b shows the variations in knee joint trajectories for the same subject at different walking speeds and for different subjects at the same walking speed, respectively.

Analysis shows that the duration of each gait phase and the amplitude of joint angle changes vary significantly among different gait trajectories. These variations can be described by several key temporal and angular points on the gait trajectory, including the initial point of the stance phase, the maximum flexion point of the stance phase (start of the stance extension phase), the end point of the stance extension phase, the maximum flexion point of the swing phase, and the end point of the swing phase, as indicated by the red dots on the curves in Figure 8. By predicting these key gait trajectory parameters, personalized knee joint trajectories that adapt to different walking speeds can be generated. Therefore, by selecting the spatiotemporal information of these key points as gait trajectory parameters and using the gait parameter prediction model to predict them, the desired gait trajectories can be generated.

The key points of the gait trajectory can be expressed as $\Theta_{via}={\left\{\theta_{n}^{via},t_{n}^{via}\right\}}_{n=1}^{N}$, where *N* represents the number of key points, including the initial point of the stance phase, the initial point of the stance extension phase, the end point of the stance extension phase, the maximum flexion point of the swing phase, and the end point of the swing phase. Therefore, N=5. The final gait parameter set is defined as

(4) $$G=\left[\Theta_{via1},\Theta_{via2},\Theta_{via3},\Theta_{via4},\Theta_{via5}\right]$$

**Figure 10:**
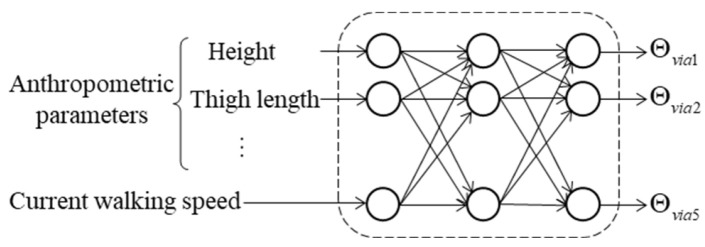
Gait parameter prediction model structure.

Thus, the set *G* contains 10 gait parameters in total. As shown in Figure 10, the gait parameter prediction model is established based on a three-layer perceptron. Considering that the input of the gait parameter prediction model includes seven anthropometric parameters and the current walking speed, while the output includes ten gait parameters, the number of nodes in the input layer, output layer, and hidden layer are set to 8, 10, and 64, respectively. The Adam algorithm is used to train the model parameters.

To evaluate subject-level generalization and reduce the risk of overfitting, the gait parameter prediction model was trained and validated using a subject-wise cross-validation strategy based on the healthy-subject gait database. A subject-wise 5-fold cross-validation scheme was adopted, in which the 25 subjects were partitioned into five folds (five subjects per fold). In each fold, data from 20 subjects were used for model training, while data from the remaining 5 unseen subjects were used exclusively for testing. This procedure ensures that gait cycles from the same subject do not appear simultaneously in both training and testing sets, thereby providing a strict evaluation of subject-level generalization. The reported prediction performance was obtained by averaging the test results across all five folds. In addition, a learning-curve analysis was conducted to further assess the robustness of the prediction model with respect to training-set size. The model was trained using increasing numbers of training subjects (e.g., 5, 10, 15, and 20 subjects), while evaluation was performed on held-out unseen subjects using a subject-wise split. For each training-set size, subjects were randomly sampled multiple times, and the prediction performance was summarized as the mean ± standard deviation.

### 3.3. Mapping Method Between Walking Speed and Gait Parameter Prediction Model

When different amputees wear the prosthetic knee joint, the anthropometric parameters of the sound side are first measured and input into the PC-based host computer. These parameters are then transmitted to the prosthesis controller via Bluetooth communication and used as the input of the gait parameter prediction model.

In order to generate the desired knee joint trajectory that varies with walking speed during locomotion, it is necessary to identify the current walking speed, which serves as another input to the gait parameter prediction model. According to the correlation analysis between walking speed and the duration of the stance flexion phase, walking speed is found to be highly negatively correlated with the stance flexion phase duration, with a correlation coefficient of −0.730. Therefore, in actual prosthesis control, once the anthropometric parameters are determined, the system can retrieve the gait trajectory key-point data corresponding to discrete walking speed levels. The stance flexion phase duration (i.e., the gait parameter $t_{2}^{via}$) is used to represent the current walking speed.

In the established gait parameter model, the number of walking speed levels ${\left\{V_{y}\right\}}_{y=1}^{Y}$ is Y=9, covering speeds from 2.0 km/h to 6.0 km/h with an interval of 0.5 km/h. The mapping relationship between the walking speed $V_{Y}$ and the stance flexion phase duration $t_{2}^{act}$ is expressed as follows:

(5) $$\left\{\begin{array}{l} V_{c}=V_{1},\;if\;t_{2}^{act}\in \left({\left\{t_{2}^{via}\right\}}_{V_{1}},\frac{{\left\{t_{2}^{via}\right\}}_{V_{1}}+{\left\{t_{2}^{via}\right\}}_{V_{2}}}{2}\right)\\ V_{c}=V_{k},\;if\;t_{2}^{act}\in \left(\frac{{\left\{t_{2}^{via}\right\}}_{V_{k-1}}+{\left\{t_{2}^{via}\right\}}_{V_{k}}}{2},\frac{{\left\{t_{2}^{via}\right\}}_{V_{k}}+{\left\{t_{2}^{via}\right\}}_{V_{k+1}}}{2}\right)\\ V_{c}=V_{9},\;if\;t_{2}^{act}\in \left[\frac{{\left\{t_{2}^{via}\right\}}_{V_{8}}+{\left\{t_{2}^{via}\right\}}_{V_{9}}}{2},{\left\{t_{2}^{via}\right\}}_{V_{9}}\right] \end{array}\right.$$

where ${\left\{t_{2}^{via}\right\}}_{V_{y}}$ is the stance flexion phase duration at walking speed $V_{y}$, $V_{c}$ is the current walking speed, and $k=\left\{2,\;3,\;\ldots ,\;8\right\}$. During actual walking, when the gait phase detection system detects the heel-strike and foot-flat events, the duration of the stance flexion phase can be measured. Based on this duration, the current walking speed level can be mapped, and the ideal knee joint motion trajectory can then be obtained from the gait parameter prediction model.

### 3.4. Swing Phase Damping Adjustment Based on Fuzzy Logic Control

**Figure 11:**
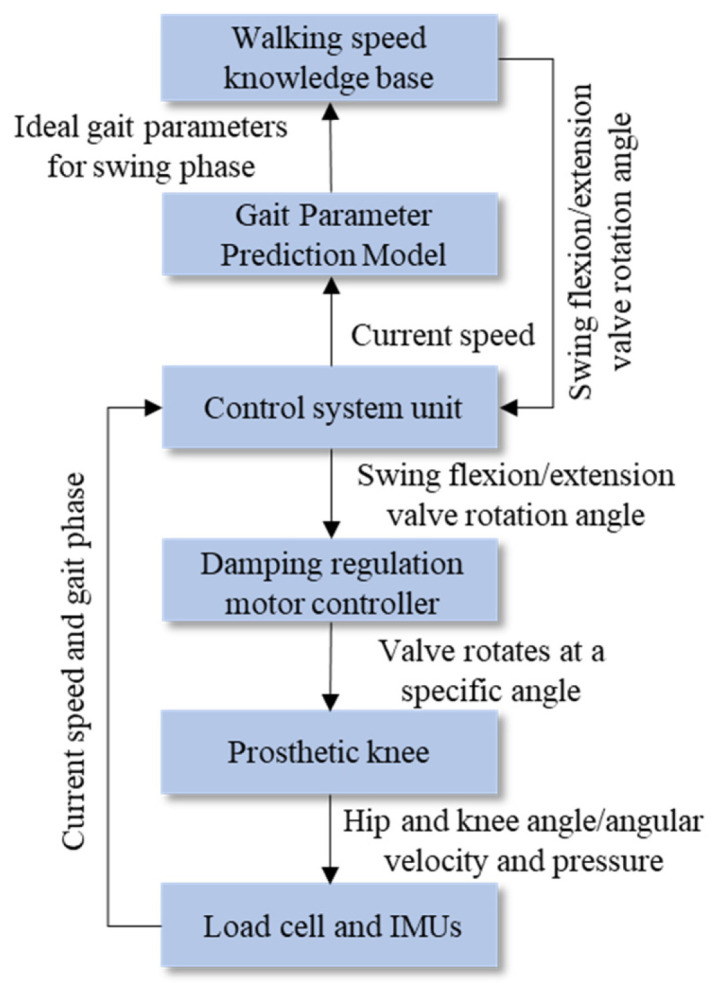
Adaptive damping adjustment scheme for the prosthetic knee during the swing phase.

Based on the proposed hybrid active–passive driving rule for different gait phases, the control objective during the swing phase is to adjust the flexion and extension hydraulic damping so that the key points of the prosthetic knee trajectory fit the corresponding ideal knee trajectory key points. Specifically, the maximum flexion point in the swing phase should match the target key point $\Theta_{via4}$, and the end point of the swing phase should match the target key point $\Theta_{via5}$. Therefore, it is necessary to establish a control method for the valve rotation angle in the hydraulic flow regulation module. When the current walking speed is detected and the key gait trajectory points $\Theta_{via4}$ and $\Theta_{via5}$ are predicted, the valve rotation angle must be adjusted to the appropriate position to output the proper flexion or extension damping, thereby realizing personalized speed-adaptive control. To achieve this objective, this study proposes a fuzzy logic–based swing phase damping adaptive control scheme, as shown in Figure 11.

According to the above analysis and the proposed swing phase damping adaptive adjustment scheme, it is essential to establish a personalized walking-speed knowledge base that contains the valve rotation angle information for swing flexion and swing extension. These angles support the prosthetic knee in fitting the gait trajectory key points under different walking speeds. The amputee–prosthesis coupling system is an unstable nonlinear system. Fuzzy logic control can achieve good control performance in nonlinear systems through simple fuzzy inference [29]. Therefore, a fuzzy logic control algorithm suitable for adaptive damping adjustment during the swing phase is proposed. The core idea of this algorithm is to compare the difference between the actual gait trajectory key point parameters and the ideal gait trajectory key point parameters during the swing phase, and to adjust the valve rotation angle online accordingly. Meanwhile, the walking-speed knowledge base can be iteratively updated to improve adaptability and control accuracy.

**Figure 12:**
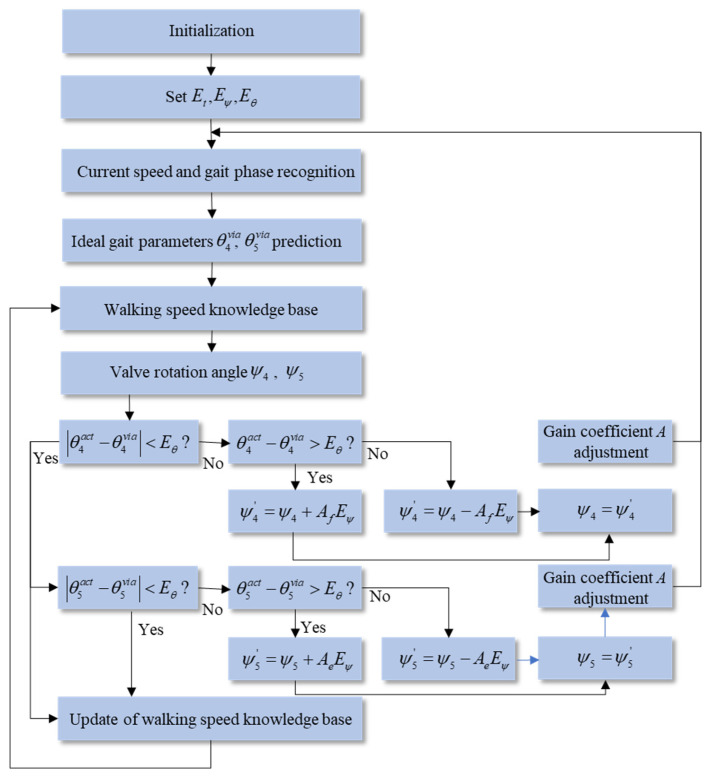
Speed-adaptive control algorithm based on fuzzy logic.

The algorithm control framework is shown in Figure 12. In the figure, $t_{4,5}^{via}$ represents the predicted time values of the 4th and 5th key points in the ideal gait parameters, that is, the time values of the maximum flexion point and the end point of the swing phase. $t_{2,4,5}^{act}$ represents the measured time values, including the duration of the stance flexion phase and the time values of the maximum flexion point and the end point of the swing phase. $\theta_{4,5}^{via}$ denotes the predicted joint angle values of the maximum flexion point and the end point of the swing phase in the ideal gait parameters. $\theta_{4,5}^{act}$ denotes the measured joint angle values of the maximum flexion point and the end point of the swing phase. $E_{t}=\frac{1}{80}\cdot t_{5}^{via}$ is the time error threshold, and $E_{\theta }$ is the joint angle error threshold. $E_{\psi }=5^{\circ }$ represents the minimum adjustment value of the valve rotation angle, and A is the gain coefficient.

**Figure 13:**
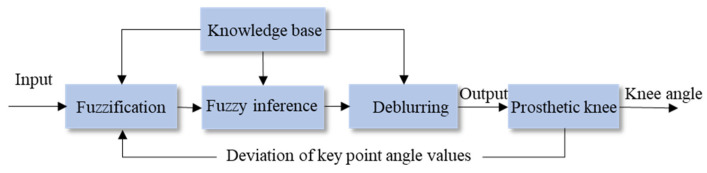
Structure of the fuzzy control system.

The gain coefficient *A* is adjusted through fuzzy logic control. When the error value increases, the gain coefficient is increased to improve the convergence speed; when the error value decreases, the gain coefficient is reduced to ensure the stability of the control system and achieve rapid convergence. Based on fuzzy control, this algorithm evaluates and feeds back the control performance according to the degree to which the swing phase gait key points fit the ideal gait key points. A PD algorithm is employed to modify the coefficient *A* obtained from fuzzy inference. Consequently, the valve rotation angle corresponding to the ideal gait parameters at the current walking speed is obtained, forming a closed-loop control to enhance the control performance. The structure of the fuzzy control system is shown in Figure 13.

The inputs of this algorithm are the key point angle deviation value *E* between the previous and the current gait cycles, and the change rate of the deviation value ΔE. The output is the gain coefficient *A*. Based on this algorithm, a simplified walking-speed knowledge base for one subject was obtained, as shown in Table 3.

The walking speed and the valve rotation angle show a positive correlation, which is consistent with the theoretical values derived from the hydraulic flow regulation module model of the prosthetic knee joint. The fuzzy control combined with the PD algorithm provides fast convergence, resulting in a shorter time required to establish the walking-speed knowledge base. Moreover, the knowledge base can be continuously updated and learned during subsequent walking. In addition, this control method requires fewer iterations, enabling efficient implementation on a microcontroller.

### 3.5. Stance Phase Assistive Extension Based on Position–Torque Control

**Figure 14:**
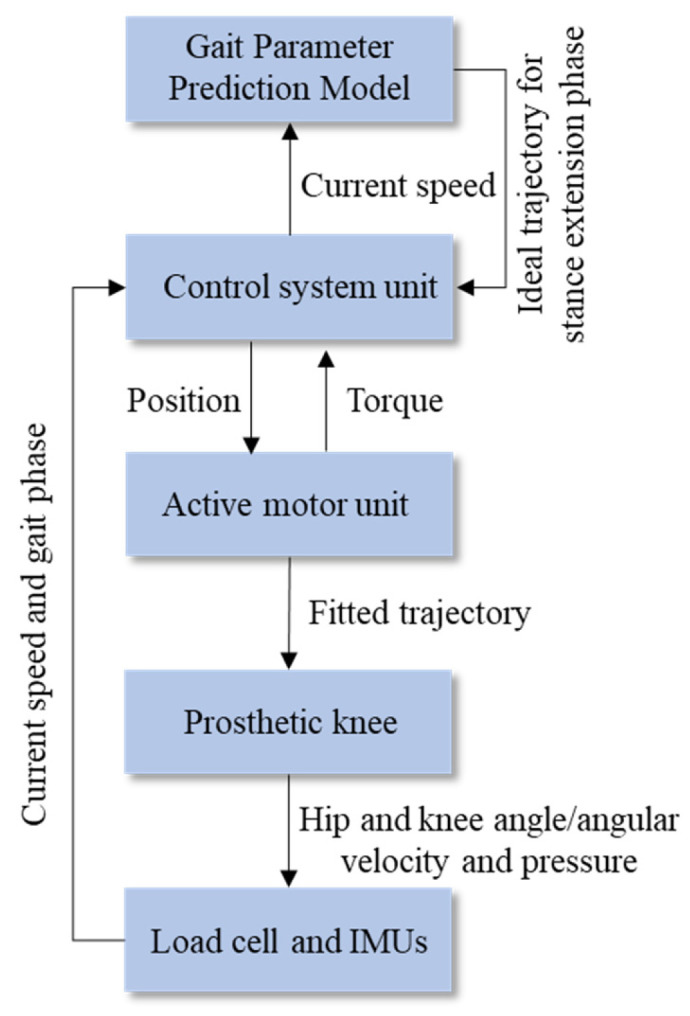
Flowchart of stance extension control based on position–torque control.

Based on the proposed hybrid active–passive driving rule for different gait phases, during the stance extension phase, the prosthetic knee joint needs to provide an active driving torque to assist knee extension. When the system identifies that the current gait phase has entered the stance extension phase, the active driving motor is activated to rotate, driving the lower piston upward through the synchronous belt and ball screw transmission mechanism, thereby providing assistive torque for knee extension. Considering that after entering the pre-swing phase, the knee joint begins to flex in preparation for the swing phase, the lower piston must quickly return to its bottom position at this moment to achieve rapid decoupling between the active driving module and the hydraulic damping module. In this study, position–torque control is employed to make the prosthetic knee joint track the knee motion trajectory during stance extension. The knee joint trajectory in the stance extension phase is approximated as a straight line connecting the starting point $\Theta_{via2}$ and the ending point $\Theta_{via3}$ of the stance extension phase. The control process of assistive knee extension based on position–torque control is shown in Figure 14.

When the amputee walks with the prosthetic knee joint, once the system recognizes that the gait has entered the stance extension phase, the parameters of the key points $\Theta_{via2}$ and $\Theta_{via3}$ in the stance extension phase can be predicted according to the mapping between the stance flexion phase duration and walking speed. The ideal knee trajectory during the stance extension phase is then obtained by linear fitting between these key points. At this moment, the prosthesis is quickly controlled to provide active driving torque. When the measured output torque of the active driving motor approaches the no-load condition, it indicates that the amputee is about to complete the stance extension and enter the pre-swing phase. The motor is then controlled to quickly reverse, driving the lower piston to return to its bottom position, thereby achieving torque decoupling and preventing interference with knee motion during the pre-swing phase.

## 4. Experiments and Results

### 4.1. Experiment Protocol

To evaluate the performance of the proposed personalized speed-adaptive control method, variable-speed treadmill walking experiments were conducted with three unilateral transfemoral amputee participants under two control strategies: (1) the proposed gait-parameter-prediction-based adaptive control, and (2) the traditional fixed-gait control without adaptive adjustment. Participant characteristics are summarized in Table 4. Each subject walked at three steady-state speeds—slow (2.5 km/h), medium (3.5 km/h), and fast (4.5 km/h)—using the same hybrid active–passive prosthetic knee prototype. For comparison, the experiments were repeated on the same prosthesis using a non–speed-adaptive fixed-gait control strategy. In this case, the knee joint was hydraulically locked during the stance phase to ensure stability, while a fixed swing flexion target of 65° was applied across all walking speeds during the swing phase, which is a common control approach for passive intelligent prosthetic knees [30]. For clinical deployment, calibration for a new user can follow a practical prosthetist workflow: (i) measure the required anthropometric parameters, (ii) initialize the speed-conditioned reference gait parameters/trajectory using the prediction model, and (iii) complete routine prosthesis alignment and safety checks prior to walking.

**Figure 15:**
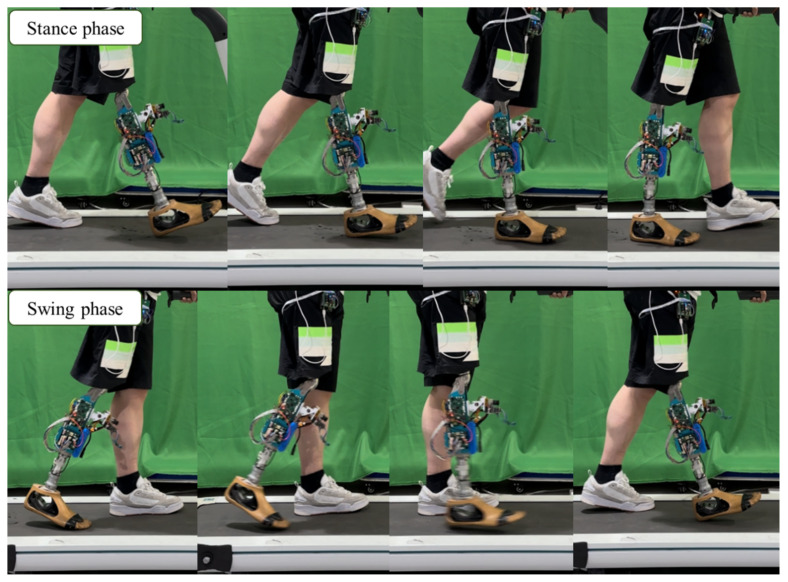
Variable-speed walking experiment protocol.

Before testing, all subjects underwent one week of gait training (2–2.5 h per day) under the supervision of a certified prosthetist, until they could walk comfortably on the treadmill. During the experiments, a motion analysis system (Ulltium, Noraxon Co., Ltd., USA) was used to capture bilateral knee joint angles at a sampling frequency of 100 Hz, as shown in Figure 15. For each walking speed, data were collected continuously for two minutes, and the average gait cycle from the middle one-minute segment was used for analysis. All participants provided written informed consent after being informed of the experimental procedures. The study protocol was approved by the Institutional Review Board of Shanghai University of Science and Technology Affiliated Shidong Hospital (Ref. No. IRB-AF63).

### 4.2. Gait Parameter Prediction Results

The gait parameter prediction model was trained offline using the established gait database D and anthropometric parameter database H. Its predictive performance and robustness were first evaluated at the model-development stage using subject-wise cross-validation and learning-curve analysis, before being applied to amputee walking experiments. Using the subject-wise 5-fold cross-validation scheme, the prediction accuracy was evaluated on unseen subjects in each fold, as shown in Table 5. Across the five folds, the predicted gait parameters showed good agreement with the measured values, with consistent performance across subjects and walking speeds. The cross-validation results indicate that the model generalizes well at the subject level and does not rely on memorization of individual gait patterns.

**Figure 16:**
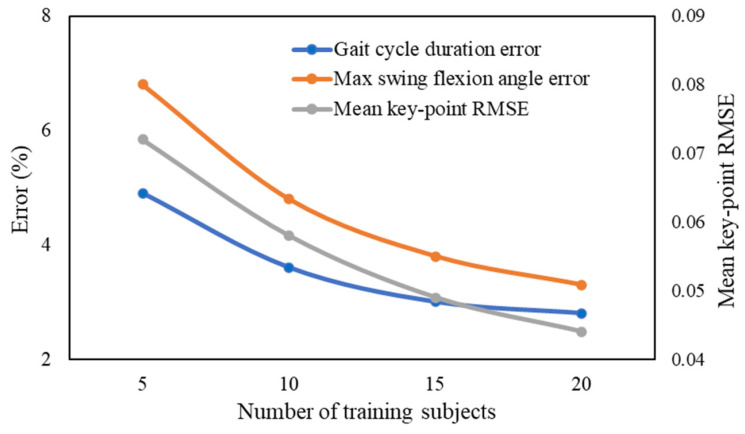
Learning-curve results with increasing number of training subjects.

In addition, a learning-curve analysis was conducted to assess the influence of training-set size on prediction performance, as shown in Figure 16. As the number of training subjects increased, the prediction error gradually decreased and stabilized, demonstrating that the model performance improves systematically with additional data and does not exhibit signs of severe overfitting when trained on a limited number of subjects. Together, the cross-validation and learning-curve results confirm the robustness of the proposed perceptron-based gait parameter prediction model for low-dimensional gait-parameter regression.

**Figure 17:**
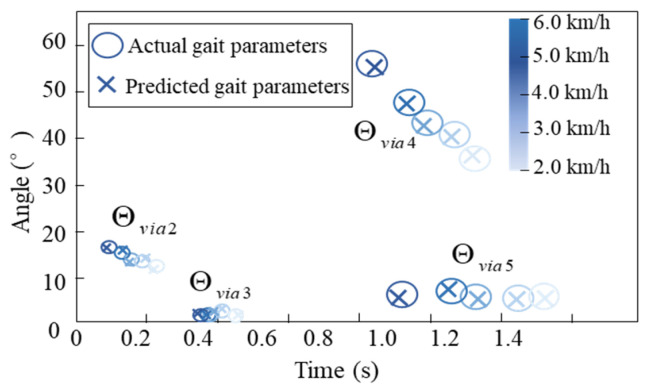
Predicted gait parameters of one representative amputee subject at different walking speeds.

After offline training and validation, the gait parameter prediction model was integrated into the prosthetic knee control framework and evaluated during walking experiments with transfemoral amputee participants. Figure 17 shows the predicted gait parameters for one representative amputee subject at different walking speeds, where each parameter value represents the mean of 50 steady-state gait cycles. The results demonstrate that the predicted gait key-point parameters—including the timing and angular values of stance and swing phases—closely match the experimentally measured values across all walking speeds. The average prediction error for gait cycle duration was within 2.8%, and for the maximum swing flexion angle within 3.5%, confirming that the proposed perceptron-based model effectively captures the nonlinear mapping between anthropometric features, walking speed, and gait parameters.

### 4.3. Experimental Results of Variable-Speed Walking

**Figure 18:**
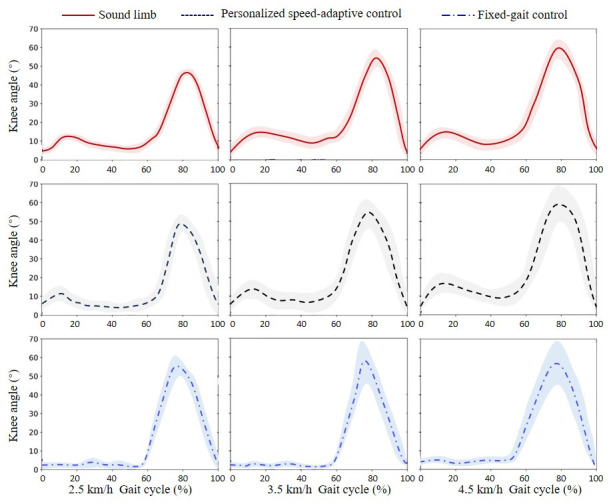
Results of the variable-speed walking experiment on level ground.

The experimental results are presented in Figure 18. Under the proposed adaptive control strategy, the prosthetic knee joint trajectory exhibited a closer match to that of the sound limb across all walking speeds, with smooth transitions between stance and swing phases. The stance flexion and extension were continuous and natural, while the swing phase trajectory remained consistent with the contralateral limb, demonstrating effective adaptation to variations in walking speed. In contrast, with the fixed-gait control strategy, the prosthetic knee failed to adjust its motion according to speed changes. Although overall motion stability was maintained, the angular deviation from the sound limb was greater at both slow and fast speeds. The absence of stance flexion reduced shock absorption during weight acceptance, and the swing phase trajectory showed a noticeably larger deviation from the contralateral limb, reflecting limited adaptability to dynamic gait variations.

**Figure 19:**
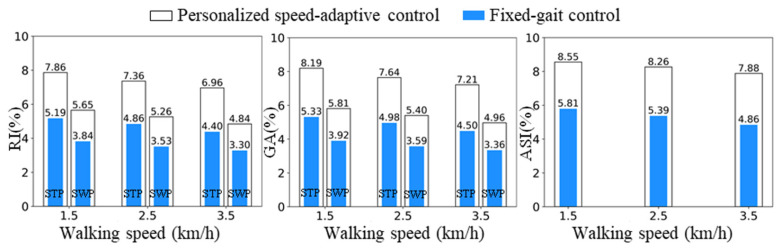
Quantitative results of gait symmetry indices. STP = stance phase, SWP = swing phase.

To provide a more intuitive and quantitative evaluation of the improvement in gait symmetry, three symmetry indices were adopted: the Ratio Index (RI) and Gait Asymmetry (GA) to quantify stance-phase and swing-phase symmetry, respectively, and the Absolute Symmetry Index (ASI) to evaluate overall gait symmetry throughout the gait cycle [31].

(6) $$RI=\left|1-\frac{X_{P}}{X_{H}}\right|\times 100\%$$

(7) $$GA=\ln\left|\frac{X_{P}}{X_{H}}\right|\times 100\%$$

(8) $$ASI=\left|\frac{2(X_{H}-X_{P})}{X_{H}+X_{P}}\right|\times 100\%$$

where $X_{P}$ and $X_{H}$ denote the value of the gait variable *X* measured from the prosthetic limb and the sound limb, respectively. In this study, *X* is the phase duration, and therefore *RI* and *GA* are computed separately for both the stance phase (STP) and the swing phase (SWP) by using the corresponding stance/swing duration in Equations (6) and (7). The ASI in Equation (8) is computed using the full gait-cycle duration to quantify overall symmetry across the entire gait cycle. When *RI* = 0, *GA* = 0, and *ASI* = 0, the gait is considered perfectly symmetric. The quantitative results are shown in Figure 19.

Across different walking speeds, the proposed personalized speed-adaptive control achieved reductions of 33.96–36.78% in RI and 34.92–37.58% in GA during the stance phase, indicating improved stability and symmetry through continuous adjustment of damping and assistive torque. During the swing phase, RI and GA decreased by 31.81–32.88% and 32.25–33.51%, respectively, demonstrating more accurate trajectory tracking and smoother swing recovery. The overall ASI of the gait cycle decreased by 32.04–38.32%, confirming that the proposed control method enables personalized, natural, and speed-consistent gait patterns. To complement the percentage-based comparison, paired statistical comparisons between the personalized and fixed control strategies were conducted across subjects. Table 6 reports the mean ± SD, paired mean differences, 95% confidence intervals, and *p*-values for RI, GA, and ASI, providing statistical support for the observed symmetry improvements.

We additionally reported the controller’s transient performance using response time and settling time computed from the logged signals during experiments. The response time was defined as the time required for the controlled variable to reach 90% of its final change after a reference/parameter update, and the settling time was defined as the time for the tracking error to enter and remain within a tolerance band of ±2°. Based on the logs, the average response time and settling time were 0.09 s and 0.22 s, respectively.

## 5. Discussion

### 5.1. Effectiveness of Personalized Gait Prediction

The gait parameter prediction model successfully captured the nonlinear relationships between anthropometric parameters, walking speed, and knee motion features. The low average prediction errors (within 2.8% for gait cycle duration and 3.5% for swing-phase maximum flexion angle) indicate that even with a limited training dataset, the perceptron-based model generalizes well to unseen subjects. This capability demonstrates the feasibility of predicting gait characteristics for new amputees using easily measurable body parameters, avoiding repeated gait calibration or manual parameter tuning commonly required in conventional systems.

### 5.2. Performance of Adaptive Control Strategy

The experimental results confirmed that the adaptive control strategy significantly enhanced prosthesis–human coordination. By dynamically adjusting hydraulic damping during the swing phase and providing active torque during stance extension, the prosthesis achieved gait trajectories more consistent with those of the sound limb. Quantitative symmetry analyses revealed that the Ratio Index (RI), Gait Asymmetry (GA), and Absolute Symmetry Index (ASI) decreased by approximately 30–38% under the proposed control, confirming marked improvements in gait stability and continuity. These findings suggest that personalized adaptive control can effectively bridge the gap between fixed-parameter prostheses and user-specific biomechanical needs. While kinematics and symmetry indices provide objective gait-quality indicators, we acknowledge that they do not directly capture patient-centered outcomes such as comfort, perceived effort, metabolic energy cost, and satisfaction, which will be addressed in future clinical studies.

In addition to knee kinematics and symmetry indices, the interaction between the prosthesis and the residuum–socket interface plays an important role in shaping overall gait biomechanics and user experience. The proposed speed-adaptive control strategy leads to smoother knee motion and more consistent phase transitions across walking speeds, which may indirectly influence how loads are transmitted through the socket to the residual limb.

Specifically, improved knee trajectory tracking and reduced asymmetry can help mitigate abrupt changes in joint torque and angular velocity, particularly during stance-to-swing and swing-to-stance transitions. Such reductions in dynamic discontinuities may lower transient interface load peaks at the residuum–socket interface, potentially decreasing localized pressure and shear stresses experienced by the user. From a biomechanical perspective, more natural and speed-consistent knee motion may also promote more symmetric whole-body gait patterns, reducing compensatory strategies at the hip and trunk that are often associated with elevated socket loading and discomfort.

Although socket pressure and shear were not directly measured in the present study, the observed improvements in knee motion smoothness and gait symmetry suggest a plausible mechanism by which the proposed control approach could contribute to improved socket tolerance and walking comfort. These considerations provide important context for interpreting the experimental results and motivate future studies that explicitly quantify socket-interface loading and patient-reported comfort outcomes alongside kinematic and symmetry metrics.

### 5.3. Comparison with Previous Studies

Compared with previous adaptive control approaches that relied on fixed thresholds [16,17,18], phase-based damping control [19,20,21,22], or fuzzy/neural-network models trained on single-subject data [23,24,25,26], the proposed method provides improved personalization and adaptability. Similar to discrete speed switching strategies, the proposed controller also divides the walking speed range into several levels. However, unlike conventional methods that use the same preset gait parameters for all users, the proposed system generates individualized gait parameter mappings for each subject based on their anthropometric characteristics and current walking speed. This enables personalized trajectory fitting within each speed level, allowing the prosthesis to better accommodate user-specific biomechanical variations.

Furthermore, the integration of a fuzzy–PD control scheme allows real-time adjustment of valve rotation angles and iterative refinement of the speed–damping relationship. Consequently, the system achieves adaptive modulation of both damping and assistive torque in each gait phase, improving smoothness and coordination compared to fixed-parameter prostheses.

### 5.4. Limitations and Future Work

Although the proposed method achieved substantial improvements, several limitations remain. First, the gait database was built from a limited number of healthy subjects and covered only level-ground walking, which may limit external validity when transferring the learned gait patterns to diverse transfemoral amputee biomechanics (e.g., compensatory strategies and inter-subject variability). Expanding the dataset to include slopes, stairs, and uneven terrains will enhance model robustness, and future evaluations should further include non-steady locomotion conditions such as start/stop, speed transitions, and turning to assess generalization beyond steady-state walking. Second, the perceptron model, while effective, was adopted for its computational simplicity and suitability for stable real-time deployment and straightforward personalization on the embedded controller, but it does not explicitly capture longer-range temporal dependencies across gait cycles, especially under transient and non-steady conditions; future work could incorporate recurrent or transformer-based neural architectures to model cycle-to-cycle temporal dynamics and contextual history for improved prediction accuracy.

Third, although the proposed control framework was validated in laboratory walking trials with a small cohort of transfemoral amputees, broader clinical validation across larger populations and longer-term daily life use is still required, including users with different functional levels and socket conditions. Beyond calibration, practical challenges related to clinical deployment—such as device cost, prosthetist training, and long-term maintenance—may also affect real-world adoption and should be considered when translating the proposed approach into routine clinical practice.

In addition, future work will integrate dynamic and neuromuscular cues (e.g., EMG) and wearable sensing to further improve subject-specific adaptation (see Section 3.2), thereby strengthening cross-user generalization in real-world settings. It should also be noted that data-driven personalization inherently depends on the representativeness of the training dataset; limited population diversity may introduce algorithmic bias, potentially reducing effectiveness for users with different ages, activity levels, or amputation etiologies. Moreover, the accessibility of advanced prosthetic systems may be constrained by cost, availability of specialized clinical support, and infrastructure requirements, which are important considerations for equitable deployment.

From a translational perspective, smoother knee motion and phase transitions may help mitigate transient interface load peaks and improve comfort; therefore, future studies will quantify socket-interface loading (e.g., pressure/shear) together with patient-reported outcomes (e.g., comfort, perceived stability, and satisfaction) and physiological measures such as metabolic/energy cost. In addition, future work will incorporate safety-related outcomes (e.g., stability metrics and fall-risk–relevant measures) and longer-term daily life evaluation to assess real-world usability and clinical benefit. Finally, the integration of on-board adaptive learning could enable the prosthesis to continuously refine control parameters during daily use, potentially reducing manual tuning during clinical deployment and achieving lifelong personalized adaptation.

## 6. Conclusions

This study proposed a personalized gait parameter prediction–based speed-adaptive control method for a hybrid active–passive prosthetic knee. By integrating anthropometric information and walking speed into a perceptron-based gait prediction model, the system can generate individualized gait parameters and achieve adaptive modulation of damping and assistive torque across different gait phases. Experimental results demonstrated that, compared with traditional fixed-gait control, the proposed method enables smoother motion transitions, more natural knee trajectories, and a 30–38% improvement in gait symmetry indices (RI, GA, ASI). These findings verify the feasibility and effectiveness of the proposed approach for realizing personalized, speed-adaptive, and natural gait control in intelligent prosthetic knees.

## Figures and Tables

**Table 1:** The mechatronic characteristics of the HAPK.

Characteristics	Values
DC motor in AMU	Maxon EC-4pole 30
Timing belt transmission	44:22 teeth ratio
Ball screw lead	1 mm
DC motor in HDU	Maxon DCX 10L with 16:1 gear stage
Maximum knee torque	30 Nm
Knee range of motion	0~135°
Weight	2865 g
Length ^1^	400~460 mm

^1^ Weight and length include lower leg tube and prosthetic foot.

**Table 2:** Subjects’ anthropometric parameters for gait database construction.

No.	Anthropometric Parameter (cm)	Mean (cm)	Standard Deviation (cm)
1	Height	172.14	6.21
2	Thigh length	43.47	2.29
3	Shank length	41.15	1.86
4	Ankle height	6.95	0.79
5	Foot length	25.09	0.85
6	Maximum thigh circumference	48.84	3.98
7	Maximum shank circumference	36.02	2.14

**Table 3:** Mapping relationship between gait characteristics and valve rotation angle.

Walking Speed (km/h)	Stance Flexion Duration (ms)	Max Swing Flexion Angle (°)	Heel-Strike Angle (°)	Valve Angle (°)	
				Swing Flexion	Swing Extension
2.0	669~750	44	5	CCW ^1^ 20.2	CW 13.6
2.5	582~669	46	6	CCW 21.1	CW 14.5
3.0	529~582	50	6	CCW 22.1	CW 14.9
3.5	450~529	53	7	CCW 22.9	CW 15.6
4.0	390~450	55	7	CCW 23.8	CW 16.1
4.5	342~390	56	8	CCW 25.1	CW 16.9
5.0	272~342	56	8	CCW 26.7	CW 18.0
5.5	209~272	57	9	CCW 28.3	CW 19.1
6.0	120~209	58	9	CCW 29.1	CW 20.2

^1^ CCW = counterclockwise rotation; CW = clockwise rotation. Valve angles denote the commanded rotation relative to the initial (0°) valve position shown in Figure 3 (minimum damping). CCW/CW indicates the rotation direction from this reference.

**Table 4:** Transfemoral amputee participant information.

Participant	A1	A2	A3
Gender	Male	Male	Female
Age (year)	32	44	27
Height (cm)	178	175	163
Thigh length (cm)	44.3	44.1	43
Shank length (cm)	42.7	41.8	37.7
Ankle height (cm)	7.2	7.1	6.4
Foot length (cm)	26.1	25.9	23.5
Maximum thigh circumference (cm)	51.1	50.2	47.3
Maximum shank circumference (cm)	38.3	36.9	34.6

**Table 5:** Subject-wise 5-fold cross-validation results for gait parameter prediction.

Fold	Gait cycle Duration Error (%)	Max Swing Flexion Angle Error (°)	Mean Key-Point RMSE (Normalized)
1	2.6	3.2	0.041
2	2.9	3.6	0.044
3	2.7	3.4	0.043
4	3.1	3.8	0.047
5	2.8	3.5	0.045
Mean ± SD	2.82 ± 0.18	3.50 ± 0.22	0.044 ± 0.002

**Table 6:** Paired statistical comparison of symmetry metrics between Personalized and Fixed control across speeds and gait phases.

Metric	Speed (km/h)	Phase	Personalized (Mean ± SD)	Fixed (Mean ± SD)	Mean Diff (Pers-Fixed)	95% CI of Diff	*p*-Value
RI	1.5	STP	7.86 ± 0.95	5.19 ± 0.88	2.67	[−1.30, 6.64]	0.1
	2.5	STP	7.36 ± 0.90	4.86 ± 0.92	2.5	[−0.98, 5.98]	0.09
	3.5	STP	6.96 ± 1.05	4.40 ± 0.96	2.56	[−0.67, 5.79]	0.08
	1.5	SWP	5.65 ± 0.85	3.84 ± 0.90	1.81	[−1.17, 4.79]	0.12
	2.5	SWP	5.26 ± 0.78	3.53 ± 0.82	1.73	[−1.00, 4.46]	0.11
	3.5	SWP	4.84 ± 0.80	3.30 ± 0.77	1.54	[−0.94, 4.02]	0.12
GA	1.5	STP	8.19 ± 1.10	5.33 ± 1.00	2.86	[−0.12, 5.84]	0.054
	2.5	STP	7.64 ± 1.00	4.98 ± 0.95	2.66	[−0.07, 5.39]	0.053
	3.5	STP	7.21 ± 0.95	4.50 ± 0.90	2.71	[0.23, 5.19]	0.043
	1.5	SWP	5.81 ± 0.90	3.92 ± 0.85	1.89	[−0.59, 4.37]	0.08
	2.5	SWP	5.40 ± 0.85	3.59 ± 0.80	1.81	[−0.43, 4.05]	0.07
	3.5	SWP	4.96 ± 0.88	3.36 ± 0.86	1.6	[−0.51, 3.71]	0.08
ASI	1.5	All	8.55 ± 1.00	5.81 ± 0.95	2.74	[0.26, 5.22]	0.042
	2.5	All	8.26 ± 1.05	5.39 ± 0.98	2.87	[0.14, 5.60]	0.046
	3.5	All	7.88 ± 1.10	4.86 ± 1.05	3.02	[−0.21, 6.25]	0.056

## Data Availability

The data supporting this study’s findings are available upon reasonable request from the authors.
